# Comparison of 3D Sensors for Automating Bolt-Tightening Operations in the Automotive Industry

**DOI:** 10.3390/s23094310

**Published:** 2023-04-27

**Authors:** Joana Dias, Pedro Simões, Nuno Soares, Carlos M. Costa, Marcelo R. Petry, Germano Veiga, Luís F. Rocha

**Affiliations:** 1 INESC TEC—INESC Technology and Science, 4200-465 Porto, Portugal; 2 Europneumaq—Soluções Industriais, 4410-052 Serzedo, Portugal; 3 Faculty of Engineering, University of Porto (FEUP), 4200-465 Porto, Portugal

**Keywords:** 3D perception, 3D sensors comparison, assembly automation

## Abstract

Machine vision systems are widely used in assembly lines for providing sensing abilities to robots to allow them to handle dynamic environments. This paper presents a comparison of 3D sensors for evaluating which one is best suited for usage in a machine vision system for robotic fastening operations within an automotive assembly line. The perception system is necessary for taking into account the position uncertainty that arises from the vehicles being transported in an aerial conveyor. Three sensors with different working principles were compared, namely laser triangulation (SICK TriSpector1030), structured light with sequential stripe patterns (Photoneo PhoXi S) and structured light with infrared speckle pattern (Asus Xtion Pro Live). The accuracy of the sensors was measured by computing the root mean square error (RMSE) of the point cloud registrations between their scans and two types of reference point clouds, namely, CAD files and 3D sensor scans. Overall, the RMSE was lower when using sensor scans, with the SICK TriSpector1030 achieving the best results (0.25 mm ± 0.03 mm), the Photoneo PhoXi S having the intermediate performance (0.49 mm ± 0.14 mm) and the Asus Xtion Pro Live obtaining the higher RMSE (1.01 mm ± 0.11 mm). Considering the use case requirements, the final machine vision system relied on the SICK TriSpector1030 sensor and was integrated with a collaborative robot, which was successfully deployed in an vehicle assembly line, achieving 94% success in 53,400 screwing operations.

## 1. Introduction

Smart manufacturing began when the vision of Industry 4.0 expanded the efficiency and flexibility expectations of automation by emphasizing on process digitalization. Now, over a decade later and witnessing the digital transformation in industries and our shared challenges in society, a new paradigm shift is changing the expectations from the industries to go beyond efficiency and accept their role as sustainable service providers for society.

The worldwide automotive industry is already progressing toward the Industry 5.0 transformation. They are, in fact, one of the leading sectors in terms of the adoption of new technologies, seeking to combine human expertise with the capabilities of intelligent machines to improve manufacturing processes and empower workers, increasing product personalization, while retaining or even enhancing their quality. This desire for rapid adoption stems from the automakers’ and suppliers’ needs to quickly respond to changes in market demand, resulting in increasingly customized products with shorter lead times.

To achieve these goals, collaborative robotic solutions are one of the most important technologies of Industry 4.0 and 5.0. They allow for the creation of innovative solutions to automate manufacturing processes and provide flexibility to the production system. Using machine vision, these robots can achieve new levels of autonomy by being able to understand shapes, calculate volumes, track objects and pack boxes with minimal wasted space, while, at the same time, enabling dynamic interactions with their human partner [1].

The introduction of machine vision in robotic systems is usually motivated by its potential to reduce costs by improving efficiency and productivity, reduce errors, enhance production quality, fill the gaps caused by labor shortages, and gather production data. Improvements to worker health and safety are also critical benefits of applying robotics and machine vision to industrial operations. Robots with machine vision can take over dull, dirty and dangerous industrial activities, as well as interpreting human actions and interacting to help prevent accidents before they happen [2,3].

Depending on the industrial context, however, the use of machine vision systems is not always trivial. Several factors may hinder their performance, such as the presence of textureless surfaces, perspective distortion, adverse lighting conditions, photometric variations, and moving objects, among many others [4]. Over the past years, the demand for machine vision solutions that can overcome these problems has increased significantly. This demand led to the increase of machine vision solutions comprised of three-dimensional (3D) optical sensors [5] since they are less sensitive to ambient lighting conditions and dirt [6].

Over time, the quality of the data provided and the variety of 3D sensors available on the market have increased as a result of the advances made in terms of sensing hardware, with more compact electronic systems with greater processing capacity and also software, with the emergence of more advanced image-processing algorithms. Moreover, advances in the quality of the data generated by 3D sensors have had a significant impact on the use of robotic systems in high-accuracy applications. As a result of these improvements, 3D sensors are being widely adopted to develop machine vision systems in many areas of R&D and industrial automation, such as mobile robot localization, obstacle detection, object recognition, pose estimation, security, human–machine interaction, and many others. Depending on the system’s requirements, various types of 3D sensors can be used [7].

Given this context, this paper presents a comparison of 3D depth sensors to determine which one is better suited for automating fastening tasks in an automotive assembly line. More specifically, the automation of bolt-tightening operations [8,9,10] during the process of fastening rear axle dampers to the undercarriage of vans will be analyzed.

This 3D sensor comparison adds to the state of the art the analysis of empirical data retrieved from three sensors under challenging industrial conditions, namely, white painted surfaces that have some degree of reflectivity. The comparison analyzed the root mean square error (RMSE) and overlap percentage between the sensor data and reference point clouds. Moreover, these metrics were analyzed for different subsampling scales using a voxel grid algorithm.

The present study offers a comparative analysis of sensors that serves to complement the metrics proposed in prior evaluations, thereby providing a more comprehensive understanding of their performance characteristics. Pinto et al. [11] compared a set of depth cameras by computing the mean distance and standard deviation of the 3D points to planar surfaces detected with a random sample consensus (RANSAC) algorithm. On the other hand, Halmetschlager-Funek et al. [12] analyzed the precision, bias and lateral noise of sensors on different lightning conditions when observing objects with different materials. Heide et al. [13] evaluated stereo cameras, using as metrics the point cloud density, the smoothness of the surface points captured from the walls, the consistency of the edges, and the mean distance between the 3D points and the ground truth surfaces along with a comparison of their surface normals. For simultaneous localization and mapping (SLAM) use cases, Neto et al. [14] relied on the mean distance between the poses estimated by the SLAM algorithm and the ground truth poses, which were known because the sensors were mounted on a robotic arm for following a pre-programmed trajectory. For the evaluation of light detection and ranging (LIDAR) sensors, Lambert et al. [15] computed the RMSE between the 3D points and the ground truth surface along with the analysis of the residual errors for assessing distance bias in the sensor measurements [16]; besides the Z-depth precision analysis using RMSE, the authors also evaluated the angle-dependent reflectivity, edge precision, spatial resolution (number of measurements per cm${}^{2}$ at a given distance), radius reconstruction accuracy and surface continuity.For visually displaying the difference between the sensor data and the surface scanning models (plane, sphere, cube, box, cylinder and dodecahedron), Chen et al. [17] also relied on color maps to complement the RMSE analysis.

Besides performing the comparison of the sensors’ performance, this paper also provides the success rate of an automated bolt-tightening machine that relies on one of the sensors under analysis. Unlike other approaches of bolt tightening that perform perception of the bolt itself [18,19,20,21], the system deployed relied on the 3D perception of the structure of the van in which the bolts were attached. This approach allowed for unambiguous 6 degrees of freedom (DoF) pose estimation, as the van’s structure has a surface with unique geometry and a higher number of points compared to the bolts. Furthermore, the proposed approach does not have the problem of ambiguity in the 6-DoF pose of the bolts due to their symmetry axes.

In the assembly line where the automated tightening machine was deployed, a van is transported by an aerial conveyor throughout different workstations, and when it arrives at the workstation under analysis, the operator picks an axle damper from a bin, goes underneath the vehicle, and installs it in the van rear undercarriage, which has two attachment points. Later on, the operator places the respective two bolts and gives them a few turns. Next, using a pantograph, the operator guides two electric screwdrivers for fastening both bolts at once. For each van, this process is performed for the left and right rear wheels. This task is repetitive and non-ergonomic, which may cause musculoskeletal injuries to the human operator. As such, the goal is to develop a collaborative robotic solution, where the operator is responsible for placing the axle damper and the bolts, and then the robot performs the fastening operations using two electric screwdrivers. For ensuring reliable operation, the collaborative robotic system must be capable of locating the axle damper attachment pose and fastening the two bolts using the two electric screwdrivers. Since the van 6-DoF pose at this specific workstation varies due to the mechanical tolerances of the aerial conveyor and weight of the vehicle, the robot needs to perceive the pose of the axle damper attachment structure in order to successfully perform the bolt-tightening operations.

The remaining of this paper is organized as follows. Section 2 presents some fundamental concepts regarding 3D sensing technology. Section 3 describes in detail the comparison of the 3D sensors within the described use case, including the methodology used for this comparison, the results obtained with each sensor and the respective discussion. Section 4 describes the integration of the sensor on the collaborative robotic solution and the assessment of its performance. Section 5 presents the conclusions of this study.

## 2. 3D Sensing Technologies

Three-dimensional sensors can be classified as active or passive according to their imaging technology [22]. Passive sensors, such as stereo cameras, rely on the light reflected from external sources for observing the environment, while active sensors rely on their own source of radiation for probing the environment, making them more robust for scanning textureless surfaces and dark environments. Examples of active sensing technologies include but are not limited to laser triangulation, structured light and time of flight.

One of the most reliable and accurate optical sensing technology is laser triangulation (point or line). The resulting point cloud 3D data are computed by interpreting the deformation of the laser line when observed from the camera perspective. Coupled with a known movement of the object on a conveyor or the sensor mounted on a track or robotic arm, several 3D scan profiles can be merged together to form a 3D point cloud of the surface to be analyzed. These sensors are usually small and have a high acquisition rate (1 kHz). The most significant disadvantage of this technology is the requirement to generate a known movement, either of the object or of the sensor itself. Despite this, 3D laser triangulation is often chosen because it provides greater robustness not only in terms of variations in ambient light but also in terms of the materials and color of the objects of interest, making it desirable in many industrial applications [23].

Structured light sensors consist of a light projector and one or more cameras [24]. The light source projects a set of known patterns into the environment, which are distorted when they hit the surface of objects. Depending on the pattern used, one or more images need to be captured. For example, a speckle pattern is static and needs only one image capture for generating sparse depth information, which can be coupled with measurements interpolation to increase the point cloud density. On the other hand, sequential stripe patterns can achieve dense surface measurements with much higher point cloud density but require several image captures with a static environment, one for each pattern with decreasing stripe thickness. The 3D sensors based on structured light are one of the most used in the industry for 3D perception and inspection tasks, given their high accuracy, high density point cloud and robustness for scanning textureless surfaces and operating in a wide range of light conditions.

Time-of-flight (ToF) cameras rely on infrared light pulses for probing the environment. They estimate the distance to objects by measuring the time difference between the pulse emission and the detection of the reflected signal [25]. The interest in these sensors has been increasing mainly due to their applicability in autonomous vehicles. Typically, these sensors have less accurate 3D data when compared to structured light sensors and generate a lot more shadow/veiling points on the border of objects, but they have a much higher data acquisition rate.

Stereo vision systems can perform 3D reconstruction of the scene by calculating the correspondence between pixels of two different images taken by cameras in different perspectives using triangulation. Since the accuracy of 3D measurements depends heavily on identifying and correctly matching points between images from different cameras, some stereo vision systems project a pattern into the environment to refine point matching. This approach significantly improves the measurement accuracy in low-texture environments. However, the consistency of the measurements is not as reliable as the previously mentioned technologies. Moreover, passive stereo systems have higher measurements errors when operating in low-light environments. These sensors are less used in industrial applications because of these limitations [26].

## 3. Comparison of 3D Sensors

### 3.1. Selection of the 3D Sensors

Different types of sensors can be used depending on the requirements of the machine vision system. The goal of the use case under analysis is to determine with high accuracy (<3 mm) the 6-DoF pose of the axle damper attachment structure, which does not have texture and is painted with partially reflective white color. Moreover, the sensor would be operating without controlled light conditions and needs to be compact to be mounted on the end effector of a robotic arm.

**Figure 1:**
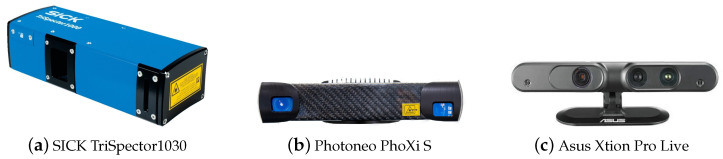
Selected 3D depth sensors.

With this use case in mind, three sensors based on active depth sensing technologies were chosen, namely, the SICK TriSpector1030 (laser line triangulation), the Photoneo PhoXi S (visible stripe pattern structured light) and the Asus Xtion Pro Live (IR speckle pattern structured light). They are presented in Figure 1, and their technical specifications are presented in Table 1.

From these three sensors, only the 3D point cloud was evaluated. The 2D image provided by the Photoneo and Asus was not considered since the goal is to estimate the 6-DoF pose of the target object. Moreover, the SICK does not provide a 2D image; it only provides 3D data.

In relation to embedded processing, only the SICK has this feature, in which the sensor is programmed using the SOPAS Engineering Tool software. The Photoneo and Asus need an external PC to process the sensor data. In order to have a fair comparison between these sensors, the embedded capabilities of the SICK sensor were not used, and the evaluation software was run in an external PC for processing the 3D data retrieved from each of the three sensors.

Other sensors were considered, such as the Intel Realsense D435 (active stereo sensor), but from our preliminary tests, it had slightly higher surface measurement error when compared to the Asus Xtion Pro Live. From the ToF sensor technology field, we also analyzed the Azure Kinect, but it had a lot of shadow/veiling points, which complicated the 3D data segmentation stage of the axle damper (even though this issue could be mitigated with the statistical outlier removal filter from the point cloud library (PCL) [27]). From the passive stereo range of sensors, the Nerian Scarlet and the Carnegie Robotics Multisense could be possible candidates, but since this passive sensing technology would not perform well in the non-textured surface of the van, we opted not to include them in the comparison.

In the end, we chose to keep the sensor comparison concise and limit the scope to the two main candidates (SICK TriSpector1030 and Photoneo PhoXi S), which were built for industrial use cases, while also including a lower cost sensor from the non-industrial/consumer marker for having a entry level sensor in the comparison results. In the future, we might consider the Ensenso N35 since it is an industrial rated sensor and from its specifications it would perform between the Photoneo PhoXi S and the Asus Xtion Pro Live.

### 3.2. Methodology for Evaluating the 3D Sensors

**Figure 2:**
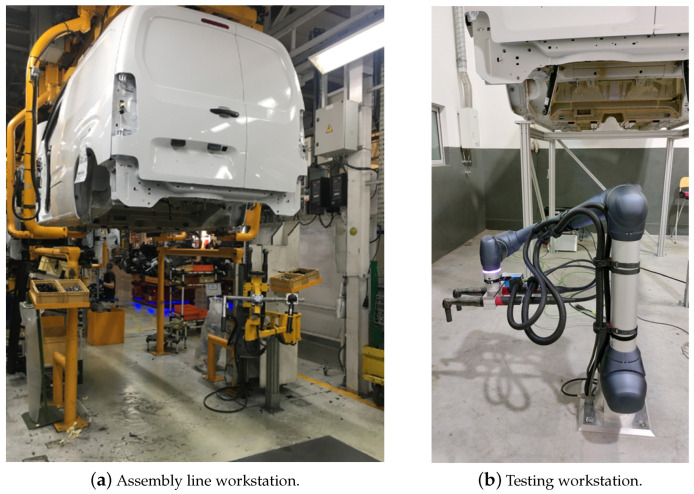
Workstations on the assembly line and the testing environment for fastening the two rear axle dampers.

To compare the performance of the three sensors, point clouds of the axle damper attachment structure with and without the axle damper were acquired in a testing environment similar to the real assembly line, namely, with a real van and axle damper samples that could be manually placed and removed (as shown in Figure 2).

**Figure 3:**
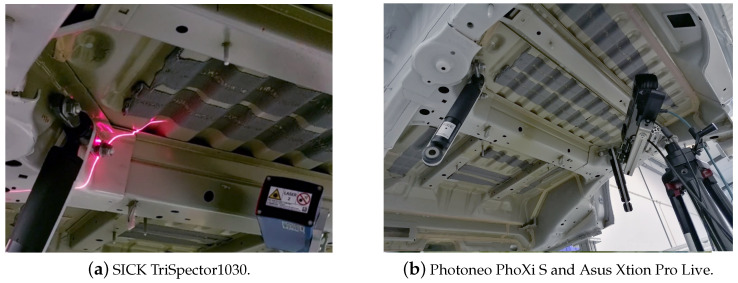
Testing workstation with the 3D sensors placed for capturing measurements.

The structured light sensors were mounted on a tripod since they need to be static during the scanning procedure. The same does not apply to the laser line triangulation sensor, which was installed on the end effector of a robotic arm (Doosan H2017) to capture the 3D profiles of the region of interest (ROI). Figure 3 shows the location of the sensors with respect to the van undercarriage.

For creating the sensor evaluation dataset, 12 scans were captured for both sides of the van with each sensor. For performing the comparison, the RMSE metric presented in the Equation (1) was used, which computes the mean distance between the sensor data points (*S*) and their respective closest point in the reference point cloud (*R*):

(1) $$RMSE=\sqrt{\frac{1}{n}\sum_{i=1}^{n}{∥S_{i}-ClosestPoint\left(R,S_{i}\right)∥}^{2}}$$

It is important to mention that in this testing workstation, the van was fixed to a rigid structure and not to an aerial conveyor (that was present in the assembly line workstation). Therefore, to simulate the aerial conveyor deviations on the van positioning, the sensors poses were slightly changed manually before performing 3D scanning.

**Figure 4:**
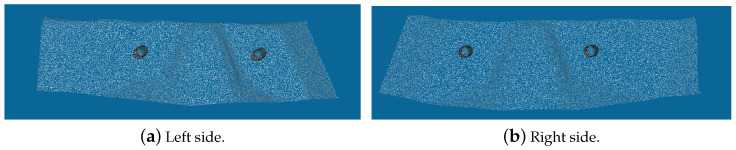
Point clouds of the ROI extracted from the van’s CAD file (without the axle damper) for each side of the van.

Two reference point clouds were used as the ground truth, one extracted from the scans without the axle dampers and another from the CAD model of the van, Figure 4. The CAD model was provided by the car manufacturing company, and a ROI was applied to extract the required surface section.

To create the reference point cloud from the 3D sensors scans, the point cloud without the axle dampers was filtered and segmented with the following steps:

1. Scan the van without the axle damper.
2. Crop the point cloud if necessary (only applicable for the Asus Xtion Pro Live sensor due to its higher scan volume).
3. Segment the point cloud into clusters using the region growing segmentation algorithm [28].
4. Extract the ROI where the axle damper will be placed (considering the acquired point clouds, this corresponds to the biggest cluster).

The region growing segmentation algorithm starts by sorting the points by their curvature and then selects as the first seed the point with the lowest curvature. Then, it keeps expanding the current cluster seeds by adding neighboring points that have an angle between the current seed normal and the neighboring point normal below a given threshold. After no more points can be added to the current cluster, a new cluster is initialized with a seed point that has the lowest curvature from the points that do not yet belong to a cluster. The algorithm for growing and creating new clusters keeps repeating until all the points are associated with a labeled cluster.

This segmentation algorithm was selected because the van support structure has a locally smooth surface with transition zones to the axle damper surfaces with large curvature differences. Moreover, the support structure has a surface area that is much higher when compared with the axle damper, allowing the segmentation selector to pick the cluster with the largest number of points.

After this procedure was executed for all the reference point clouds without the axle damper, the point clouds acquired with the axle damper were filtered by following the same steps as described above. Then, the registration of both point clouds was performed with different voxel grids (1 and 5 mm). The accuracy of the point cloud registration using the iterative closest point (ICP) algorithm [29] was measured by computing the RMSE, which was obtained by computing the Euclidean distance between corresponding points from the scan and reference point clouds. The RMSE was calculated for each registration [30], in which points with a corresponding reference point distance lower than a given threshold were marked as inliers.

The ICP algorithm aligns the sensor data with the reference point cloud by iteratively computing the 6-DoF matrix transformation that minimizes the RMSE of a given set of correspondences. For each iteration, every point in the sensor data is matched with the respective closest point in the reference point cloud. Points that have a correspondence distance higher than a given threshold are discarded from the list of correspondences to allow the algorithm to tolerate outliers. Then, the singular value decomposition (SVD) method is used to compute the 6-DoF transformation that minimizes the RMSE of the correspondences distances. The algorithm stops when the RMSE is below a given threshold or when the computed matrix has converged and stabilized, having a difference in relation to the previous iterations below specified translation and rotation thresholds. On the other hand, to bound its computation time, the algorithm can have an upper limit to its number of iterations and its maximum run time. At the end stage, the ICP algorithm returns the 6-DoF matrix that aligns and transforms the 3D sensor data into the reference point cloud, which corresponds to the sensor’s 6-DoF pose in the reference point cloud coordinate system.

The evaluation relied on the Dynamic Robot Localization perception pipeline (https://github.com/carlosmccosta/dynamic_robot_localization, accessed on 22 April 2023) for performing the point cloud registrations and computing the RMSE. The perception pipeline [31,32,33] uses filtering, segmentation and alignment algorithms from PCL and was developed with the robot operating system (ROS) [34].

**Figure 5:**
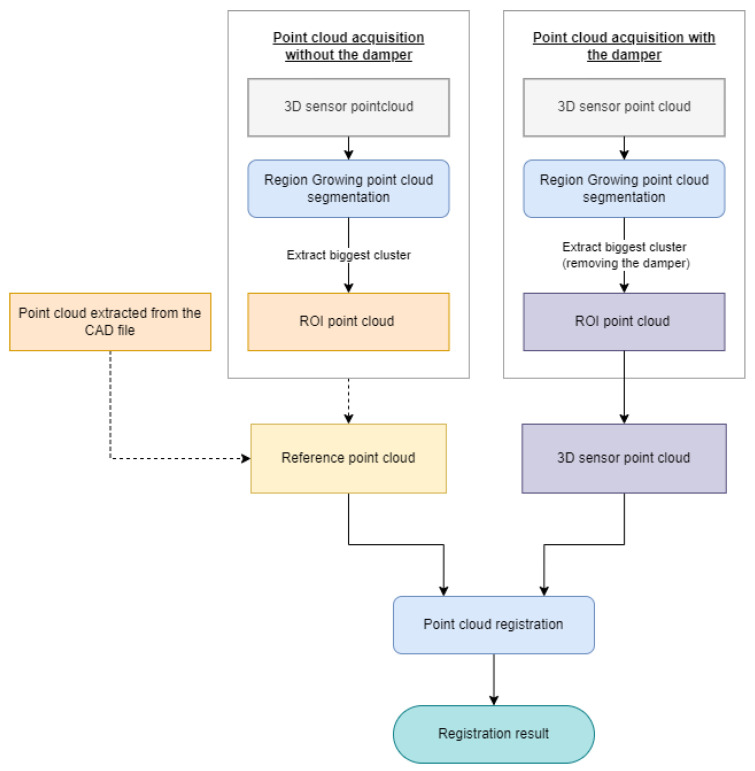
Diagram of the methodology used for comparing the different sensors.

Figure 5 summarizes the steps associated with the generation of the reference point clouds and the registration process.

### 3.3. Sensors Evaluation

**Figure 6:**
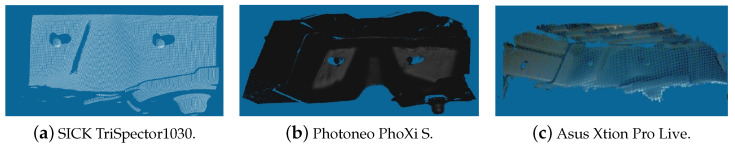
Example of the point clouds extracted from each sensor of the axle damper attachment structure (without the axle damper). It should be noted that the point cloud depicted in (**c**) is already cropped to a smaller volume.

**Figure 7:**
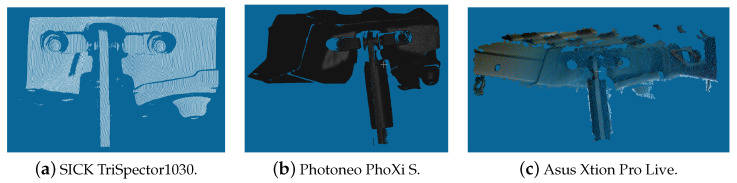
Example of the point clouds extracted from each sensor of the axle damper attachment structure (with the axle damper). It should be noted that the point cloud depicted in (**c**) is already cropped to a smaller volume.

As described in the previous section, the first step consists of multiple scans, with and without the axle damper mounted in the van. Figure 6 and Figure 7 present samples of the point clouds generated by each sensor, without and with the axle damper mounted on the van. The point clouds without the axle damper were segmented to extract only the ROI and then used as the reference point cloud for the point cloud registration.

**Figure 8:**
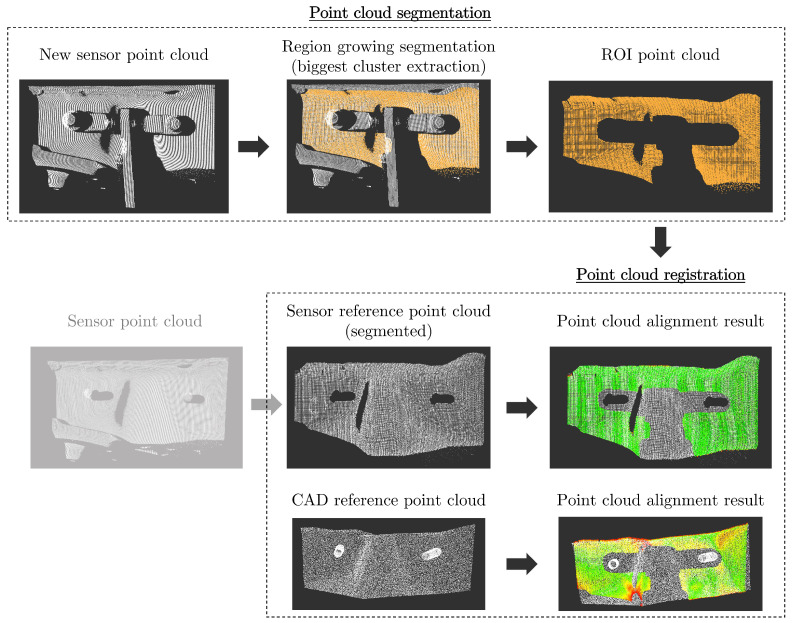
Process of segmenting and registering the point clouds (example using point clouds generated by the SICK TriSpector1030 sensor).

Figure 8 shows an example of the whole point cloud segmentation and registration process. The orange point cloud is the result of the point cloud segmentation using the region growing algorithm. In the point cloud alignment result, the color of the points are associated with the corresponding distances between the reference and the new point cloud, with green indicating that the Euclidean distance between a pair of matched points is close to zero and red indicating that the distance between the points is higher than the maximum inlier distance.

Table 2 and Table 3 summarize the registration results obtained using the sensor and the CAD model as reference point clouds, respectively, presenting the average RMSE of inliers and the average percentage of inliers in the alignment result. Although each side of the van had a different reference point cloud, resulted from a slightly different surface geometry, the assessment did not evaluate the sides separately, as the objective was to identify the sensor with the best overall results for both sides. As such, for each sensor, 12 scans were captured from the left side of the van and another 12 scans from the right side of the van. From these 24 scans for each sensor, the methodology presented in the previous subsection (with its overview in Figure 5) was used to compute the mean values and standard deviations for each metric presented in Table 2 and Table 3.

Analyzing Table 2 and Table 3, it is possible to verify that the alignment results (RMSE and inliers percentage) were better when using reference point clouds based on a previous scan performed by the respective sensor instead of using CAD models. This was expected since the production of the van structure has some deviations and tolerances in relation to the CAD model. Moreover, registering a new point cloud with a previously captured and filtered scan can be used to evaluate the repeatability of both the sensor and the alignment algorithms. On the other hand, the RMSE difference when comparing the usage of a reference point cloud using CAD models or scans is less significant when using a bigger voxel grid (5 mm) since the voxel grid replaces all the points within a cell with their mean XYZ value. This can result in the absorption of the van structure production tolerances and the sensor measurements noise but can also raise the mean RMSE if the reference and scan voxel grids do not have overlapping coordinate systems, resulting in an offset between the cells that grows as the voxel size increases.

Focusing solely on the results achieved when using the sensor-based reference point cloud and the voxel grid of 1 mm, the relative difference between the RMSE of the point clouds captured by each sensor is more clear. Namely, the lower RMSEs were 0.25 mm, 0.49 mm and 1.01 mm when using the SICK TriSpector1030, Photoneo PhoXi S and Asus Xtion Pro Live, respectively. Additionally, the percentages of inliers were 99%, 92% and 88%, respectively. Moreover, no significant difference was found when varying the maximum inlier distance.

The difference between sensors was lower when using a voxel grid of 5 mm. The lower RMSE was 1.30 mm, 1.40 mm and 1.49 mm, with a maximum inlier distance of 2 mm when using the SICK TriSpector1030, Photoneo PhoXi S and Asus Xtion Pro Live, respectively. In this case, there were significant differences when varying the maximum inlier distance. The RMSE decreases with a smaller maximum inlier distance; however, the percentage of inliers decreases as well. Although the RMSE was smaller, the value refers to a smaller number of corresponding points.

As detailed in Table 1, the depth error listed in the SICK TriSpector1030 technical specifications is smaller than the other two sensors, and this specification is reflected in these results. This sensor provides the best alignment results when using both types of reference point clouds and when varying the voxel grid and the maximum inlier distance. Considering the voxel grid of 1 mm, the RMSE was always below 1 mm with a percentage of inliers above 85% even when using the CAD point cloud as the reference model. The RMSE increased above 1 mm when changing the voxel grid for 5 mm, but, overall, the SICK TriSpector1030 sensor performed better than the other sensors.

We were also able to achieve an RMSE below 1.00 mm (around 0.50 mm) with the Photoneo PhoXi S using a sensor-based reference point cloud with a voxel grid of 1 mm. Overall, the Photoneo PhoXi S performed worse than the SICK TriSpector1030 but better than the Asus Xtion Pro Live. In general, the Asus Xtion Pro Live generated the worse results, with an RMSE always above 1 mm. This was mainly related to the lower quality of the captured point cloud, which had less accuracy and higher sensor noise.

The lower RMSE achieved by the SICK TriSpector1030 was likely due to the usage of camera lens filters that block all light with the exception of light frequencies associated with the laser line. This way, the SICK TriSpector1030 will have better repeatability since the camera sensor will have less pixel noise when compared with the other two sensors, which capture light from a much wider frequency range. On the other hand, by being a line triangulation system, SICK can also employ subpixel algorithms to estimate the center of the detected laser line, further increasing its precision and repeatability.

The time needed to process the registration and pose estimation was lower when using a bigger voxel grid (5 mm) since the point cloud was less dense (had fewer points), when compared with a point cloud generated with a smaller voxel grid (1 mm). Additionally, due to the usage of voxel grids, the density of the point clouds from each sensor was similar, which resulted in similar processing times. When considering a smaller voxel grid, there was a noticeable difference in the processing time since the density of the point clouds was higher, with a processing time proportional to the number of points registered. As described in Table 2 and Table 3, the processing time was higher when using the Photoneo PhoXi S sensor because the raw point clouds from this sensor had much higher number of points than the other sensors.

## 4. Machine Vision System for Fastening Operations

Taking into account the experimental results presented earlier and the use case requirements, such as the process cycle time limit for fastening the two axle dampers (which must be under 160 s) and the 3 mm accuracy, as well as the cost of each individual sensor, we implemented the automated axle damper fastening system with the SICK TriSpector1030 sensor. Namely, the fastening platform was equipped with the following:

1. One Doosan H2017 robotic arm with a range of 1700 mm and payload of 20 kg.
2. One SICK TriSpector1030 sensor.
3. Two screwdrivers attached to the end effector of the robotic arm.

**Figure 9:**
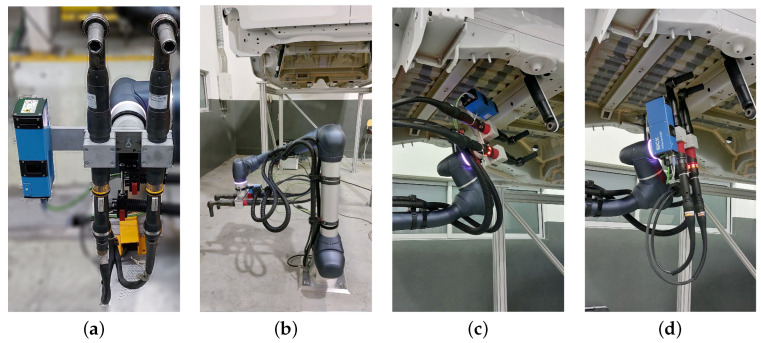
Testing environment for the automated axle damper fastening platform. (**a**) Robot tool with the 3D sensor (blue) and the two screwdrivers. (**b**) Robot with base centered with the left and right axle dampers. (**c**) Robot and tool positioned to scan the ROI. (**d**) Robot and tool positioned to perform the tightening operation.

The robot base was placed centered in relation to the left and right axle dampers locations (as seen in Figure 9) for ensuring that the platform could perform the desired fastening operations on both axle dampers without needing to move its base.

The approach implemented to determine the pose of the axle damper attachment structure was as follows:

1. Setup phase:
  (a) Robotic arm moves the 3D sensor to scan the ROI of the van without the axle damper (reference point cloud).
  (b) The pose of the attachment structure with respect to the robot is determined.
2. Operation phase performed for each side of the van:
  (a) Operator places the axle dampers on a new van.
  (b) Robotic arm moves the sensor to capture a new scan of the ROI of the van with the axle damper (seen in Figure 9c).
  (c) The sensor point cloud is aligned with the reference point cloud using the ICP algorithm.
  (d) The point cloud alignment is validated for ensuring that it has a minimum percentage overlap between the reference point cloud and the new scan.
  (e) The transformation matrix between the robot base and the axle damper attachment structure is computed.
  (f) Robotic arm moves the screwdrivers and performs the bolt-tightening operations (shown in Figure 9d).

The machine vision was deployed at the van assembly workstation at the factory, where it was tested during two weeks (split into two shifts of seven hours and twenty-five minutes each). At the screwing workstation, the vehicles stopped at 160 s. Table 4 presents the performance results obtained from these trials.

## 5. Conclusions

The usage of 3D sensors to tackle perception challenges keeps expanding since they are able to provide accurate depth information and have fewer limitations regarding the environment light conditions when compared with 2D cameras. This paper presented a comparison of three depth sensors to evaluate which one is more suitable for proving 3D data for a machine vision system that estimates the 6-DoF pose of the attachment structure of axle dampers in the undercarriage of vans. The sensor analysis was focused on comparing the suitability of the sensors when using as metrics the RMSE and overlap percentage computed after point cloud registration. For the described use case, the results indicate that the SICK TriSpector1030 is the most appropriate, given its lowest RMSE (0.25 mm ± 0.03 mm) and highest overlap percentage (99%), followed by the Photoneo PhoXi S, with RMSE (0.49 ± 0.14). On the other hand, for applications that require less accuracy and in which the low cost is an important factor, the Asus Xtion Pro live can also be a feasible option, given that it achieved an acceptable RMSE (1.01 mm ± 0.11mm). After deploying the machine vision with the SICK TriSpector1030 on an assembly line, the automated bolt-tightening system was able to achieve 94% of success in 53,400 screwing operations, with an operation time below 80 s.

Future work may include the comparison of other 3D sensors along with the inclusion of other perception algorithms that rely on fusion between 2D and 3D data for increasing the reliability of the segmentation stage for other use cases.

## Figures and Tables

**Table 1:** Technical specifications of the analyzed 3D depth sensors.

	SICK TriSpector1030	Photoneo PhoXi S	Asus Xtion Pro Live
**Category**	Laser triangulation	Sequential stripe structured light	Infrared speckle structured light
**Depth camera** **resolution**	2500 points/scan	2064 × 1544	640 × 480
**Depth camera** **field of view**	65°	40° H, 34° V	58° H, 45° V
**Depth error**	<0.04 mm	<0.05 mm	<5 mm
**Color/Grayscale** **point cloud**	Grayscale reflectance	Grayscale	Color
**Minimum** **depth**	141 mm	384 mm	800 mm
**Maximum** **depth**	541 mm	520 mm	3500 mm
**Frame rate**	5000 3D profiles/s	4 Hz	30 Hz
**Supported OS**	Linux/Windows	Linux, Windows	Linux, Windows
**Connection**	Ethernet	Ethernet	USB 2.0
**Dimensions**	217 × 62 × 84 mm	296 × 77 × 68 mm	180 × 35 × 50 mm
**Weight**	1.3 kg	900 g	100 g
**Approximated** **price**	5000 €	10,000 €	150 €

**Table 2:** Registration results using the sensor-based reference point cloud (average results for the 12 scans, providing the mean and standard deviation).

**SICK TriSpector1030**					
**Voxel Grid** **(mm)**	**Maximum Inliers** **Distance (mm)**	**RMSE** **(mm)**	**Point Cloud Size** **(Registered Points)**	**Inliers** **(%)**	**Processing** **Time (s)**
1	3	0.25±0.03	16,485±2479	99±1	8.01±1.34
1	2.5	0.26±0.03	16,485±2479	99±1	7.09±1.02
1	2	0.25±0.03	16,485±2479	99±1	8.79±1.43
5	3	1.72±0.07	1074±193	95±4	3.07±0.02
5	2.5	1.57±0.08	1074±193	86±4	3.07±0.01
5	2	1.30±0.10	1074±193	65±4	3.07±0.01
**Photoneo PhoXi S**					
**Voxel grid** **(mm)**	**Maximum inliers** **distance (mm)**	**RMSE** **(mm)**	**Point cloud size** **(registered points)**	**Inliers** **(%)**	**Processing** **time (s)**
1	3	0.52±0.17	28,434±2473	92±8	15.81±1.81
1	2.5	0.50±0.15	28,434±2473	91±10	13.49±1.41
1	2	0.49±0.14	28,434±2473	92±9	15.97±1.92
5	3	1.79±0.04	1810±151	89±9	3.11±0.01
5	2.5	1.65±0.03	1810±151	80±9	3.12±0.01
5	2	1.40±0.03	1810±151	60±8	3.12±0.01
**Asus Xtion Pro Live**					
**Voxel grid** **(mm)**	**Maximum inliers** **distance (mm)**	**RMSE** **(mm)**	**Point cloud size** **(registered points)**	**Inliers** **(%)**	**Processing** **time (s)**
1	3	1.17±0.14	10,113±3460	96±2	5.34±1.34
1	2.5	1.18±0.15	10,113±3460	80±13	5.48±1.42
1	2	1.01±0.11	10,113±3460	88±4	5.33±1.34
5	3	1.99±0.08	968±122	85±5	3.06±0.01
5	2.5	1.79±0.04	968±122	69±8	3.06±0.01
5	2	1.49±0.03	968±122	46±8	3.06±0.01

**Table 3:** Registration results using CAD-based reference point cloud (average results for the 12 scans, providing the mean and standard deviation).

**SICK TriSpector1030**					
**Voxel Grid** **(mm)**	**Maximum Inliers** **Distance (mm)**	**RMSE** **(mm)**	**Point Cloud Size** **(Registered Points)**	**Inliers** **(%)**	**Processing** **Time (s)**
1	3	0.92±0.16	16,485±2479	90±9	8.05±1.38
1	2.5	0.86±0.14	16,485±2479	89±9	7.17±1.02
1	2	0.81±0.12	16,485±2479	87±10	8.90±1.50
5	3	1.81±0.18	1074±193	83±11	3.08±0.02
5	2.5	1.68±0.14	1074±193	75±14	3.07±0.01
5	2	1.42±0.10	1074±193	54±17	3.07±0.02
**Photoneo PhoXi S**					
**Voxel grid** **(mm)**	**Maximum inliers** **distance (mm)**	**RMSE** **(mm)**	**Point cloud size** **(registered points)**	**Inliers** **(%)**	**Processing** **time (s)**
1	3	1.18±0.18	28,434±2473	72±5	15.88±1.96
1	2.5	1.12±0.17	28,434±2473	72±8	13.28±1.22
1	2	1.02±0.11	28,434±2473	66±8	16.11±1.85
5	3	2.01±0.06	1810±151	64±6	3.12±0.01
5	2.5	1.80±0.03	1810±151	52±8	3.12±0.01
5	2	1.50±0.02	1810±151	34±6	3.12±0.01
**Asus Xtion Pro Live**					
**Voxel grid** **(mm)**	**Maximum inliers** **distance (mm)**	**RMSE** **(mm)**	**Point cloud size** **(registered points)**	**Inliers** **(%)**	**Processing** **time (s)**
1	3	1.35±0.07	10,113±3460	94±2	5.45±1.39
1	2.5	1.35±0.13	10,113±3460	75±18	5.59±1.45
1	2	1.09±0.04	10,113±3460	81±4	5.45±1.39
5	3	2.05±0.02	968±122	82±5	3.06±0.01
5	2.5	1.81±0.01	968±122	64±5	3.06±0.01
5	2	1.50±0.02	968±122	40±3	3.06±0.01

**Table 4:** Performance results obtained at the car assembly workstation.

Metric	Result
Number of fastenings operations	53,400
Successful screwing operations percentage	94%
Cycle time for screwing the two axle dampers	80 s
Robot occupancy percentage	50%

## Data Availability

All data are contained within the manuscript. Raw data are available from the corresponding author upon request.
